# French Theories on Cholera

**Published:** 1884-10-20

**Authors:** 

**Affiliations:** Paris Faculty


### FRENCH THEORIES ON CHOLERA.

By M. LEREBOULLET, OF THE PARIS FACULTY.

The question of the day before the French Academy is cholera at every point of view. Among the most important communications are those on prophylaxis of the disease, by M. Noel Gueneau de Mussy, which have their foundation in the similarity of propagation of typhoid fever and cholera.

It is very evident that the French savants have keenly felt the discovery by the German professor, Koch, of a microbe, special to the disease, in the intestinal dejections of choleraic patients, after the scientific envoys of M. Pasteur to Alexandria had asserted that none such existed.

In a recent article on the subject, M. Lereboullet, of the Paris Faculty, very largely considers this part of the subject.

MM. Straus and Roux have studied at Alexandria and Toulon the pathological anatomy of cholera. They found that the lesions observed were identical in both epidemics. They have sought to discover, with the greatest care (and their competence in such research is undeniable), if there existed any microscopic organism which might be considered as the specific infectious agent of the disease, and they have failed to find any such bacillus or microbe.

Well acquainted with the works of Prof. Koch, having assisted in his researches, using the same reagents, they have, like him, found that in the intestinal dejections of individuals dying of cholera there existed a microbe of special dimensions and form. But, on the other hand, they have found that this microbe was not always present; and, again, that it could be found, not only in cholera, but also in ordinary dysenteric dejecta (malassez), in the vaginal mucus of women suffering from leucorrhœa, and in the uterine secretions of a woman having epithelioma of the os.

These observations would seem to prove that the exterior form of a microbe is not sufficient alone to characterize it, and that in the absence of direct proof, which might result from a fertile inoculation, the affirmation of M. Koch must be received with a certain amount of reserve. One fact is of very great importance, and that is the very rapid multiplication and constant appearance of these bacilli in the intestinal mucus of subjects dying very suddenly from cholera.

If, as has been asserted, a large number of these microbes be found in the intestinal mucus rapidly taken from the subject just after death from the disease, and if these special microbes described by Prof. Koch are also found later in the course of the malady, it may be possible that they constitute the infectious agent in the disease.

But, whatever be the value of this pathologic discovery, it must be admitted that, of all the maladies possessing elements of contagion or infection, cholera, in its modes of evolution and transmission, appears most compatible with the hypothesis of causation by an animated germ or microbe.

If it be proven later that Prof. Koch has been mistaken as to the form and nature of the bacillus described by him, it is probable that some other observer may discover a characteristic element remaining fertile after several cultures.

What remains demonstrated, and has even been admitted by M. Jules Guerin, is the transmissibility of the malady, or, in other words, its contagiousness, a fact established by a number of well-authenticated observations.

But if, as has been admitted, cholera is transmissible from the sick to those in health, it is no less proven that the elements of contagion are contained in the choleraic dejections; that is, in products elaborated in the intestine, rich in infectious germs, and, whether or not the doctrine of Pettenkofer be admitted, susceptible of reproduction in the soil where such dejections are deposited. After such reproduction, these germs indubitably acquire greater virulence and transmit through drinking water and by means of alimentary products, on which they may be deposited.

If the truth of these assertions be recognized, it appears difficult to admit any points of resemblance between this very grave epidemic disease and the cases of sporadic cholera or cholera morbus which occur during the hot months. M. Ernest Besnier has presented a very clear expose of this part of the subject.

At a clinical point of view, cholera morbus, or cholera nostras, as it is sometimes called, may present, in certain very exceptional cases, some analogy with Asiatic cholera; but it never kills, within a short period of time a large number of individuals, as does the other. From 1875 to 1883 it caused but one hundred and twenty deaths at Paris out of a population of two million and three hundred thousand; that is, only about fourteen each year. Again, this summer cholera attacks at the same time many cities more or less distant from each other: it does not invade successively one city after another where individuals have arrived, in apparent health, from the city first affected.

Epidemic cholera breaks out suddenly in a city after the arrival of a vessel from an infected port, or soon after the introduction of germs imported by individuals coming from a city already attacked by the epidemic.

Epidemic cholera, causing in a short space of time numerous deaths, has never been spontaneously developed in a city far from the seaboard, like Paris, Vienna or Berlin. In every epidemic the malady has raged for a certain length of time in the seaports before appearing in these central cities. The fact that the disease seems to appear simultaneously in different parts of the city does not prove that it was not originally the result of contagion.

As regards the present epidemic, the cholera which first took root in one quarter of Toulon (La Division) did not appear at Marseilles until after the arrival in the latter city of individuals emigrating from Toulon, and at the commencement of the epidemic the first cases were traced to two places from which they originally started. The same observation was often made in anterior epidemics. It will suffice to prove this to read the instructive memoir contributed by M. Jules Worms to the Gazette Hebdomadaire in 1865. But, even when the direct filiation of all the cases related cannot be established, a few negative cases will not suffice to offset the great number of positive facts which demonstrate that the epidemics follow one on the other, and that it was possible in 1865, as in the preceding epidemics in 1822, in 1830 and in 1846, to follow, step by step, the progress and extension of cholera.

M. Jules Guerin has very ably attempted to demonstrate that cholera is often developed spontaneously in the large cities, where it appears in the same manner as summer cholera; but, if he considers the history of anterior epidemics, he will find it difficult to escape the fact that their course and importation from city to city has been distinctly traced; and if he recognizes this extension as occurring in any one epidemic from one place to another, how can he reconcile this fact with the doctrine he has attempted to demonstrate of the identity of epidemic cholera with the cholera morbus of the hot months.

We repeat what we have already said regarding the very rapid progress of the disease which has swept away so many victims at Toulon and Marseilles. "If it be admitted that the choleraic germ is developed in the intestines and transmitted by the dejections, it can be very readily understood how different quarters of the same city may appear to become infected at the same moment. It is generally admitted that at the debut of the epidemic some of the individuals affected are at first, during the period of prodromic or premonitory diarrhœa, able to be about and attend to their usual occupations. By such patients several quarters of a city might be almost simultaneously infected through the water-closets and sewers into which their choleraic discharges find their way. Another source of rapid spread of the disease would be by provisions coming from infected houses.

"If it be necessary to reject, in part, the telluric theory advanced by Pettenkofer, as it explains neither the epidemics developed on board ships nor the transmission of cholera by clothing and merchandise, still he may be in the right when he asserts that the choleraic germ, in order to become noxious and infectious, has to undergo certain transformations similar to those observed in animals of alternating generations. This doctrine, if true, would explain why direct inoculation of the disease has been attempted in vain. It explains, also, the sterility of the germs collected by Koch and cultivated in liquids, perhaps unfavorable to this transformation."

Perhaps this theory would account for the fact that the cholera seems to disappear in Provence and in Italy without attacking the large cities, which are better cared for, at a hygienic point of view, and therefore do not offer a soil so favorable for the production and propagation of the infectious germs.

We have already remarked, when citing the proportion of deaths in anterior epidemics, that, like most exotic diseases, which seem to gradually become extinguished in places which have been several times attacked, cholera diminishes in gravity. The epidemic of 1873, which was certainly imported from without, disappeared after attacking several cities of Normandy and Paris, where it caused but eight hundred and fifty-five deaths. It is to be hoped that such may be the case for the present epidemic. That the disease has not already attaeked Paris and other northern cities, notwithstanding the numerous ways of communication and the absence of propyplactic measures, must undoubtedly be due to the vastly improved hygienic conditions in all the large towns.—*Med. and Surg. Rep.*

